# Body Mass Index and Depressive Symptoms in Older Adults: A Cross-Lagged Panel Analysis

**DOI:** 10.1371/journal.pone.0114891

**Published:** 2014-12-11

**Authors:** Jinseok Kim, Jin-Won Noh, Jumin Park, Young Dae Kwon

**Affiliations:** 1 Department of Social Welfare, Seoul Women's University, Seoul, Korea; 2 Department of Healthcare Management, Eulji University, Seongnam, Korea; 3 University of Maryland School of Nursing, Baltimore, Maryland, United States of America; 4 Department of Humanities and Social Medicine, College of Medicine and Catholic Institute for Healthcare Management, the Catholic University of Korea, Seoul, Korea; McMaster University, Canada

## Abstract

**Background:**

There are conflicting results about the association between body mass index (BMI) and depressive symptoms in older adults. The present study examined the relationship between weight and depressive symptoms over time in older adults in South Korea.

**Methods:**

We used data from three waves of the Korean Longitudinal Study of Aging and ran a series of cross-lagged panel models to test the reciprocal relationship between depressive symptoms and obesity in older Korean adults. We assumed a temporally stable relationship between depressive symptoms and obesity and, thus imposed equality constraints over time.

**Results:**

After controlling for the effect of depressive symptoms two years prior, underweight older adults had a higher depressive symptom score than those of normal weight. When controlling for obesity status from two years prior, older adults with higher levels of depressive symptoms were more likely to be underweight and less likely to be overweight than normal weight. The same patterns were observed in data from 2006 to 2008 and from 2008 to 2010.

**Conclusions:**

These results show that there is a correlation between depressive symptoms and weight status. In middle-aged and elderly Asian populations, depression can lead to weight loss rather than obesity, and underweight may develop depressive symptoms.

## Introduction

Obesity and depression are increasingly prevalent public health concerns [1]–[3]. The prevalence of obesity has nearly doubled worldwide since 1980 [4]. Obesity is a major risk factor for non-communicable diseases such as cardiovascular disease, diabetes, musculoskeletal disorders, and cancer [4]. Depression is estimated to affect 350 million people. The 2011 World Mental Health Survey, which included data from 17 countries, found that approximately 1 in 20 people report experiencing a depressive episode [5]. Depressive symptoms can cause considerable impairments in an individual's ability to handle daily responsibilities and can even lead to suicide [5]. In this respect, both depression and obesity can increase the burden of disease with enormous economic costs [4], [5].

A growing body of literature has investigated the relationship between body weight and depression, with controversial results. Some studies report a positive association [6]–[9], while other studies did not observe any association [10], [11], and still others report an inverse association between weight and depression [12]–[17]. Furthermore, most of the empirical evidence involved the general population, with few studies focused on weight and depression in the elderly. Since functional limitations and medical comorbidity associated with age may be related to weight and mood changes, the link between weight and depression in elderly people could be different than relationships identified in other age groups [10].

In addition, the link between weight and depression has been explored in Western populations, but in-depth investigations among Asian populations are limited. Several studies [12], [13], [15], [17] in Asian countries reported an inverse relationship between body weight and depression in the elderly. This differs from studies in Western countries, which have found a positive association between body weight and mental illness. These differences between Western and Asian countries may be due to cultural influences. There is a strong stigma of obesity in Western countries [18], yet fatness is traditionally associated with happiness in Asian countries since only wealthy people can afford to eat more and gain weight [12], [13], [17].

The majority of previous studies were limited to a single point in time when identifying the association between weight and depression. It is difficult to explore a causal relationship between two variables with cross-sectional data analysis. Longitudinal studies are important because they provide more information on the causal direction of the association, which can contribute to the development of prevention and intervention strategies. Furthermore, most of the existing studies focused on overweight individuals, substantiating the need for a survey that includes both overweight and underweight individuals. The present study categorized individuals as either overweight, obese, normal weight, or underweight, based on body mass index (BMI). The purpose of this study was to evaluate the relationship between depressive symptoms and weight over time in a large representative sample of older Korean adults.

## Methods

### 1. Data and Subjects

We used data from the Korean Longitudinal Study of Aging (KLoSA) conducted by the Korean Labor Institute and funded by the Korean Ministry of Labor, which included adults aged 45 years and older. KLoSA collected panel data from the same individuals followed through time at intervals of two years. Data from 2006 (N = 10,254), 2008 (N = 8,688), and 2010 (N = 7,920) were used in the present study. The sampling frame of KLoSA comprises enumeration districts, as identified by the National Statistical Office's 2005 Census. KLoSA, like the Health and Retirement Study (HRS) in the USA, the English Longitudinal Study of Ageing (ELSA) in the UK and the Survey of Health, Ageing and Retirement in Europe (SHARE), serves as a benchmark tool to analyze the panel data of the middle aged and elderly population. Its major research components include family, health, employment, income, asset and subjective awareness.

This study was approved by the Institutional Review Board of the Catholic University of Korea with a waiver for informed consent because the data were obtained from a public database (http://survey.keis.or.kr/USBBSGO00N.do?mnucd=OldDwn5).

### 2. Variables

The presence of depressive symptoms was assessed by the Korean version of the Center for Epidemiologic Studies Depression Scale (CES-D) survey [19]. A simplified form of the CES-D, the CES-D 10 is a questionnaire composed of 10 questions and was developed as a depressive symptom screening scale for epidemiological investigations. In the Korean version of the CES-D 10, participants were asked how frequently they experienced depressive symptoms during the past week, with four possible answers: 0 =  ‘rarely’ (less than one day); 1 =  ‘sometimes’ (from one to two days); 2 =  ‘often’ (from three to four days); 3 =  ‘at all times’ (from five to seven days). Depression score was obtained by calculating the total score of the 10 items, which ranged from 0 to 30, with a higher score indicating severe depressive symptoms. The internal consistency reliability of the depression scores as measured by Cronbach's alpha ranged from 0.822 to 0.861 across the study period.

BMI was defined as weight in kilograms divided by the height in meters squared (kg/m^2^). BMI values were calculated with self-reported weight and height. In this study, participants were classified as underweight, normal weight, overweight, or obese based on World Health Organization (WHO) Western Pacific Region suggested revised Asia-Pacific criteria (less than 18.5 kg/m^2^, between 18.5 kg/m^2^ and 23 kg/m^2^, between 23 kg/m^2^ and 25 kg/m^2^, and more than 25 kg/m^2^, respectively) [20].

### 3. Statistical Analysis

**Figure 1:**
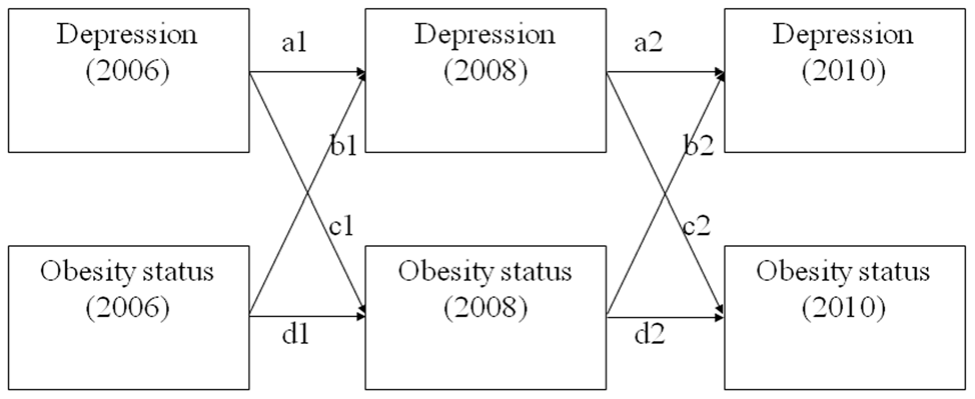
Cross-lagged panel model of depressive symptoms and obesity status.

In order to test the potentially reciprocal relationship between depressive symptoms and obesity status among older adults, we ran a series of cross-lagged panel models using data collected from three different time points with a two-year interval between each time point. Cross-lagged panel model is presented in Fig. 1 in which each variable is regressed on its own lagged score and the lagged score of the other variable in the model as well. Cross-lagged panel models are advantageous because they statistically control for all other constructs measured at the same time point. Cross-lagged analysis makes it possible to infer the underlying processes of reciprocal causality between depression and obesity [21]. In the model shown in Fig. 1, paths denoted as a and d represent the autoregressive relationship of depression and weight, respectively, and b and c represent the cross-lagged relationships between weight and depressive symptoms. In addition, suffixes 1 and 2, following the path names, were added to distinguish different time points. The stability of autoregressive and cross-lagged paths were tested by comparing the models with and without equality constraints across different time points (i.e., a1 = a2; b1 = b2; c1 = c2; d1 = d2) (Fig. 1). Separate analysis models controlling for participant's socio-demographic variables including gender, age, education level (0 =  lower than high school graduate; 1 =  high school graduate or higher), employment status (0 =  unemployed; 1 =  employed), and income were analyzed and compared to results from models without controls. Stata, version 13.1, software (StataCorp LP, College Station, Texas) was used to calculate model parameters.

## Results

Sample characteristics in terms of depressive symptoms and obesity status are summarized in Table 1. Level of depressive symptoms measured by CES-D 10 scores ranged from 16.65 (SD = 5.13) in 2006 to 17.53 (SD = 5.63) in 2010. Obesity status did not change substantially across the study period. Almost half were normal weight, followed by overweight and obese. Fewer than 5% of the participants were underweight (Table 1).

**Table 1:** Sample characteristics of depressive symptom and weight status.

	2006 (n = 10,254)		2008 (n = 8,688)		2010 (n = 7,920)	
	Mean	(SD)	Mean	(SD)	Mean	(SD)
Depressive symptoms: CES-D 10 (range: 10–40)	16.65	(5.13)	17.45	(5.66)	17.53	(5.64)
Age (years)	61.71	(11.13)	63.66	(10.92)	66.27	(10.55)
Yearly income (million KRW)	19.03	(24.79)	24.98	(28.30)	24.69	(22.30)
						
	n	(%)	n	(%)	n	(%)
Wight status						
Underweight	446	(4.46)	362	(4.26)	349	(4.52)
Normal	4,374	(43.78)	3,794	(44.62)	3,382	(43.78)
Overweight	2,884	(28.87)	2,536	(29.83)	2,225	(28.80)
Obese	2,287	(22.89)	1,810	(21.29)	1,769	(22.90)
Gender: female	5,792	(56.49)	4,921	(56.64)	4,508	(56.92)
Education: high school graduate or higher	3,765	(36.75)	3,150	(36.27)	2,866	(36.20)
Employed: now	3,888	(37.92)	3,703	(42.62)	3,397	(42.89)

CES-D: Center for Epidemiologic Studies Depression Scale; KRW, Korean won; SD: standard deviation.

Cross-lagged panel models of depressive symptoms and weight status after controlling for respondents’ socio-demographic variables were specified to explore the reciprocal relationship between depressive symptoms and weight from 2006 to 2010 with two-year intervals (Fig. 1). We assumed that the relationships between the two variables were stable over time, so we imposed equality constraints to the autoregressive paths (i.e., a1 = a2; d1 = d2 in Fig. 1) and cross-lagged paths (i.e., b1 = b2; c1 = c2 in Fig. 1). The model fit difference between the models with and without the equality constraints (i.e., a1 = a2; b1 = b2; c1 = c2; d1 = d2) was not significant [*χ* ^2^ (df = 16)  = 14.98, *p* = 0.526] suggesting that the model with equality constraints should be retained.

Table 2 summarizes the results of the cross-lagged panel model of depressive symptoms and obesity with equality constraints imposed. After controlling for the effects of depressive symptoms two years ago, underweight participants had higher levels of depressive symptoms compared to normal weight participants [B (SE)  = 1.16 (0.21), *p*<0.001]. There were no significant differences in depressive symptoms between normal weight and overweight participants [B (SE)  = −0.13 (0.10), *p* = 0.19), nor were there significant differences in depressive symptoms between normal weight and obese participants [B (SE)  = −0.03 (0.10), *p* = 0.81) (Table 2).

**Table 2:** Summary of cross-lagged panel model.

	CES-D 2008 (2010)			Underweight vs. Normal^a^			Overweight vs. Normal^a^			Obese vs. Normal^a^		
	B	SE (B)	95% CI	OR	SE (OR)	95% CI	OR	SE (OR)	95% CI	OR	SE (OR)	95% CI
CES-D 2006 (2008)	0.52***	0.01	(0.51, 0.54)	1.06***	0.01	(1.04, 1.08)	0.99*	0.00	(0.98, 1.00)	1.01	0.01	(1.00, 1.02)
BMI status 2006 (2008)												
Underweight	1.16***	0.21	(0.75, 1.57)	15.09***	1.54	(12.36, 18.42)	0.48***	0.09	(0.33, 0.69)	0.57	0.20	(0.29, 1.13)
Normal (reference)												
Overweight	−0.13	0.10	(−0.31, 0.06)	0.26***	0.06	(0.17, 0.40)	7.14***	0.33	(6.53, 7.82)	11.44***	0.75	(8.48, 11.44)
Obese	−0.03	0.10	(−0.23, 0.18)	0.42*	0.14	(0.21, 0.82)	9.20***	0.67	(7.98, 10.61)	156.67***	11.28	(112.26, 156.67)

Note: Model parameters were adjusted for gender and age, education level, employment status, and income at 2006;

*p<0.05,

**p<0.01,

***p<0.001.

CES-D: Center for Epidemiologic Studies Depression Scale; BMI: body mass index; SE: standard error; OR, odds ratio; CI: confidence interval.

a Multinomial logistic regression results were reported.

After taking into account the autoregressive effect of obesity from two years prior, subjects with a higher depressive symptom score were more likely to be underweight compared to normal weight [OR (SE)  = 1.06 (0.01), *p*<0.001] and less likely to be overweight than normal weight [OR (SE)  = 0.99 (0.00), *p* = 0.03). However, depressive symptom score was not significantly associated with the likelihood of being obese compared to being normal weight [OR (SE)  = 1.01 (0.01), *p* = 0.25] (Table 2). The same results were observed even after controlling for gender and age.

## Discussion

This study analyzed the correlation between depressive symptoms and obesity in a time series using a cross-lagged panel analysis with data from a representative group of middle aged and elderly people. Over time, depressive symptom scores were higher in the underweight group compared with the normal group; however, there was no significant difference in depressive symptom scores among the overweight or obese compared with the normal group. Over time, participants with the highest depressive symptom scores were more likely to be underweight than normal weight, and less likely to be overweight (but not obese). These results show that there is a correlation between depressive symptoms and weight status. Specifically, underweight group was likely to increase depressive symptom scores over time, and the group with a higher depressive symptom score was more likely to be underweight

Several studies have analyzed the correlation between obesity and depression in community population groups. This includes a meta-analysis using results from previous research [22], although the results of this meta-analysis are not clear. Many studies have observed a positive relationship between depression and obesity [22]–[26], while other studies report an inverse relationship or no significant relationship between the depression and obesity [27]–[29]. Drawing conclusions from these studies is difficult because results varied according to race, nation, gender, age, and time, under the control of socioeconomic status variables.

Many existing studies were limited in that they were cross-sectional analyses of obesity and depression or depressive symptoms. In contrast, the present study examined three sets of panel data at intervals of two years and evaluated the reciprocal relationship between obesity and depression. One previous study did conduct a time-sequential analysis focused on the elderly in the USA [10], and found that degree of obesity was not associated with an increase in depressive symptoms, and that depressive symptoms played a role in weight loss in men, and weight loss or gain in women. Although obesity and depressive symptoms were inversely correlated, as they are in this study, the study only observed the impact of depressive symptoms on weight (obesity), and did not observe an impact of obesity on depressive symptoms, which differs from this study. Forman-Hoffman et al. (2007) noted that the effects of depressive symptoms on weight differ by gender, and that women in particular may not just lose weight, but can also gain weight in association with depressive symptoms [10]. In a study conducted by Konttinen et al. (2014) which targeted youth in Finland at 10-year intervals, men with depressive symptoms were likely to have a higher BMI, while women with a higher BMI were likely to develop depressive symptoms [30]. Although both Konttinen et al. and Forman-Hoffman et al. observed gender differences, in Konttinen et al.'s study there was also a positive association between obesity and depressive symptoms, which differs from both our study and from Forman-Hoffman et al.'s results. It is difficult to infer because only a few studies have used time-series analyses. However, we presume the result of study can be impacted by correlations among variables such as age, nation and ethnicity.

This study used a time-sequential analysis to observe a inverse correlation between weight status and depressive symptoms in a representative sample of middle aged and elderly Koreans. In middle aged and elderly Asian populations, depression can lead to weight loss rather than obesity, and underweight may develop depressive symptoms unlike Westerners who have a positive relationship between depression and obesity. This suggests that risk-group selection in public health initiatives should be reconsidered.

This study is meaningful because it was the first to use a time-series analysis to target an Asian population. However, this study does have some limitations. It did not include all age groups, and used only BMI to measure obesity. BMI data could be under or over reported because they were self-reported. In addition, absence of information about drugs like antidepressant medications could be any effect on the observed obesity/depression relationships. And CES-D does not measure depression but depressive symptomatology. Therefore, additional research is needed to address these limitations.

## Supporting Information

S1 File List of variables and their values.
(XLS)

## References

[1] Andersen I, Thielen K, Bech P, Nygaard E, Diderichsen F (2011) Increasing prevalence of depression from 2000 to 2006. Scand J Public Health 39:857–863. 21965477 10.1177/1403494811424611

[2] Seidell JC (2005) Epidemiology of obesity. Semin Vasc Med 5:3–14. 15968575 10.1055/s-2005-871737

[3] von Ruesten A, Steffen A, Floegel A, van der A DL, Masala G, et al (2011) Trend in obesity prevalence in European adult cohort populations during follow-up since 1996 and their predictions to 2015. PLoS One 6:e27455. 22102897 10.1371/journal.pone.0027455 PMC3213129

[4] World Health Organization (2013) Obesity and overweight. Available: http://www.who.int/mediacentre/factsheets/fs311/en/

[5] Marcus M, Yasamy MT, Van Ommeren M, Chisholm D, Saxena S (2012) Depression: a global public health concern. World Health Organization paper on depression: 6–8. Available: http://www.who.int/mental_health/management/depression/who_paper_depression_wfmh_2012.pdf

[6] McCrea R, Berger Y, King M (2011) Body mass index and common mental disorders: exploring the shape of the association and its moderation by age, gender and education. Int J Obes (Lond) 36:414–421. 21427699 10.1038/ijo.2011.65

[7] Ohayon MM (2007) Epidemiology of depression and its treatment in the general population. J Psychiatr Res 41:207–213. 17113600 10.1016/j.jpsychires.2006.10.006

[8] Sachs-Ericsson N, Burns AB, Gordon KH, Eckel LA, Wonderlich SA, et al (2007) Body mass index and depressive symptoms in older adults: the moderating roles of race, sex, and socioeconomic status. Am J Geriatr Psychiatry 15:815–825. 17804833 10.1097/JGP.0b013e3180a725d6

[9] Zhong W, Cruickshanks KJ, Schubert CR, Nieto FJ, Huang GH, et al (2010) Obesity and depression symptoms in the Beaver Dam Offspring Study population. Depress Anxiety 27:846–851. 20112247 10.1002/da.20666 PMC2891299

[10] Forman-Hoffman VL, Yankey JW, Hillis SL, Wallace RB, Wolinsky FD (2007) Weight and depressive symptoms in older adults: direction of influence? J Gerontol B Psychol Sci Soc Sci 62:S43–S51. 17284566 10.1093/geronb/62.1.s43

[11] Ohayon MM, Hong SC (2006) Prevalence of major depressive disorder in the general population of South Korea. J Psychiatr Res 40:30–36. 15878179 10.1016/j.jpsychires.2005.02.003

[12] Li ZB, Ho SY, Chan WM, Ho KS, Li MP, et al (2004) Obesity and depressive symptoms in Chinese elderly. Int J Geriatr Psychiatry 19:68–74. 14716701 10.1002/gps.1040

[13] Chang HH, Yen ST (2012) Association between obesity and depression: evidence from a longitudinal sample of the elderly in Taiwan. Aging Ment Health 16:173–180. 21861766 10.1080/13607863.2011.605053

[14] Crisp AH, Queenan M, Sittampaln Y, Harris G (1980) ‘Jolly fat’ revisited. J Psychosom Res 24:233–241. 7205712 10.1016/0022-3999(80)90013-6

[15] Kuo SY, Lin KM, Chen CY, Chuang YL, Chen WJ (2011) Depression trajectories and obesity among the elderly in Taiwan. Psychol Med 41:1665–1676. 21208492 10.1017/S0033291710002473

[16] Palinkas LA, Wingard DL, Barrett-Connor E (1996) Depressive symptoms in overweight and obese older adults: a test of the “jolly fat” hypothesis. J Psychosom Res 40:59–66. 8730645 10.1016/0022-3999(95)00542-0

[17] Yu NW, Chen CY, Liu CY, Chau YL, Chang CM (2011) Association of body mass index and depressive symptoms in a Chinese community population: results from the Health Promotion Knowledge, Attitudes, and Performance Survey in Taiwan. Chang Gung Med J 34:620–627. 22196065

[18] Puhl R, Brownell KD (2003) Ways of coping with obesity stigma: review and conceptual analysis. Eat Behav 4:53–78. 15000988 10.1016/s1471-0153(02)00096-x

[19] Cho MJ, Kim KH (1998) Use of the Center for Epidemiologic Studies Depression (CES-D) Scale in Korea. J Nerv Ment Dis 186:304–10. 9612448 10.1097/00005053-199805000-00007

[20] WHO Expert Consultation (2004) Appropriate body-mass index for Asian populations and its implications for policy and intervention strategies. Lancet 363:: 157-163. 10.1016/S0140-6736(03)15268-3 14726171

[21] Shadish WR, Cook TD, Campbell DT (2002) Experimental and quasi-experimental designs for generalized causal inference. Boston: Houghton Mifflin: 506.

[22] de Wit L, Luppino F, van Straten A, Penninx B, Zitman F, et al (2010) Depression and obesity: a meta-analysis of community-based studies. Psychiatry Res 178:230–235. 20462641 10.1016/j.psychres.2009.04.015

[23] Dixon JB, Dixon ME, O'Brien PE (2003) Depression in association with severe obesity: changes with weight loss. Arch Intern Med 163:2058–2065. 14504119 10.1001/archinte.163.17.2058

[24] Dong C, Sanchez LE, Price RA (2004) Relationship of obesity to depression: a family-based study. Int J Obes Relat Metab Disord 28:790–795. 15024401 10.1038/sj.ijo.0802626

[25] Luppino FS, de Wit LM, Bouvy PF, Stijnen T, Cuijpers P, et al (2010) Overweight, obesity, and depression: a systematic review and meta-analysis of longitudinal studies. Arch Gen Psychiatry 67:220–229. 20194822 10.1001/archgenpsychiatry.2010.2

[26] Simon GE, Ludman EJ, Linde JA, Operskalski BH, Ichikawa L, et al (2008) Association between obesity and depression in middle-aged women. Gen Hosp Psychiatry 30:32–39. 18164938 10.1016/j.genhosppsych.2007.09.001 PMC2675189

[27] Carpenter KM, Hasin DS, Allison DB, Faith MS (2000) Relationships between obesity and DSM-IV major depressive disorder, suicide ideation, and suicide attempts: results from a general population study. Am J Public Health 90:251–257. 10667187 10.2105/ajph.90.2.251 PMC1446144

[28] Crisp AH, McGuiness B (1976) Jolly fat: relation between obesity and psychoneurosis in general population. Br Med J 1:7–9. 1247732 10.1136/bmj.1.6000.7 PMC1638245

[29] Pine DS, Cohen P, Brook J, Coplan JD (1997) Psychiatric symptoms in adolescence as predictors of obesity in early adulthood: a longitudinal study. Am J Public Health 87:1303–1310. 9279265 10.2105/ajph.87.8.1303 PMC1381090

[30] Konttinen H, Kiviruusu O, Huurre T, Haukkala A, Aro H, et al (2014) Longitudinal associations between depressive symptoms and body mass index in a 20-year follow-up. Int J Obes (Lond) 38:668–674. 23949617 10.1038/ijo.2013.151

